# Supplementary material for: Easymap: A User-Friendly Software Package for Rapid Mapping-by-Sequencing of Point Mutations and Large Insertions
Source: Front Plant Sci. 2021 May 7;12:655286. doi: 10.3389/fpls.2021.655286 (PMC8143052; doi:10.3389/fpls.2021.655286)
Supplement: Supplementary Figure 1 — Easymap architecture overview. [file Presentation_1.PDF]

Easymap: a user-friendly software package for rapid mapping by sequencing of point mutations and large insertions

Samuel Daniel Lup, David Wilson-Sánchez,
Sergio Andreu-Sánchez, and José Luis Micol

Instituto de Bioingeniería, Universidad Miguel Hernández, Campus de Elche,
03202 Elche, Spain

Supplemental Figures

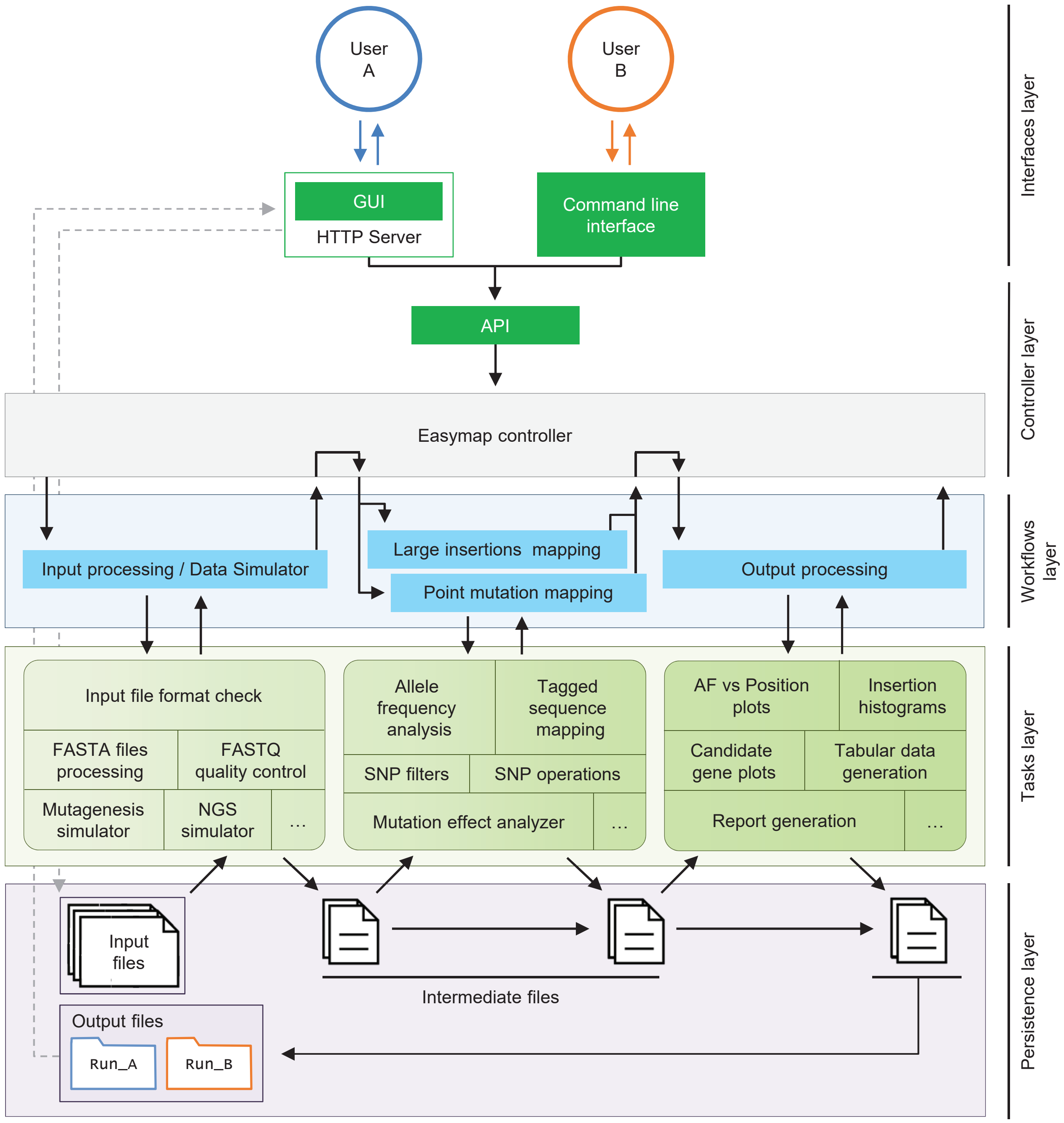

Figure S1. Easymap architecture overview.
Easymap consists of a series of layers interacting in a hierarchical manner. The Interfaces layer includes two independent modules that allow the user to control the program through a web graphical interface or a command line interface. At the Controller layer, both interfaces interact with an application programming interface (API). The Easymap controller will then execute a series of workflows from the Workflows layer that will themselves execute several custom and third-party programs to perform specific tasks during the analysis at the Tasks layer. The execution of a specific set of workflows and tasks is automated and established according to the input parameters provided through either interface. The Persistence layer represents the files being used, modified, and generated during the execution of the program.

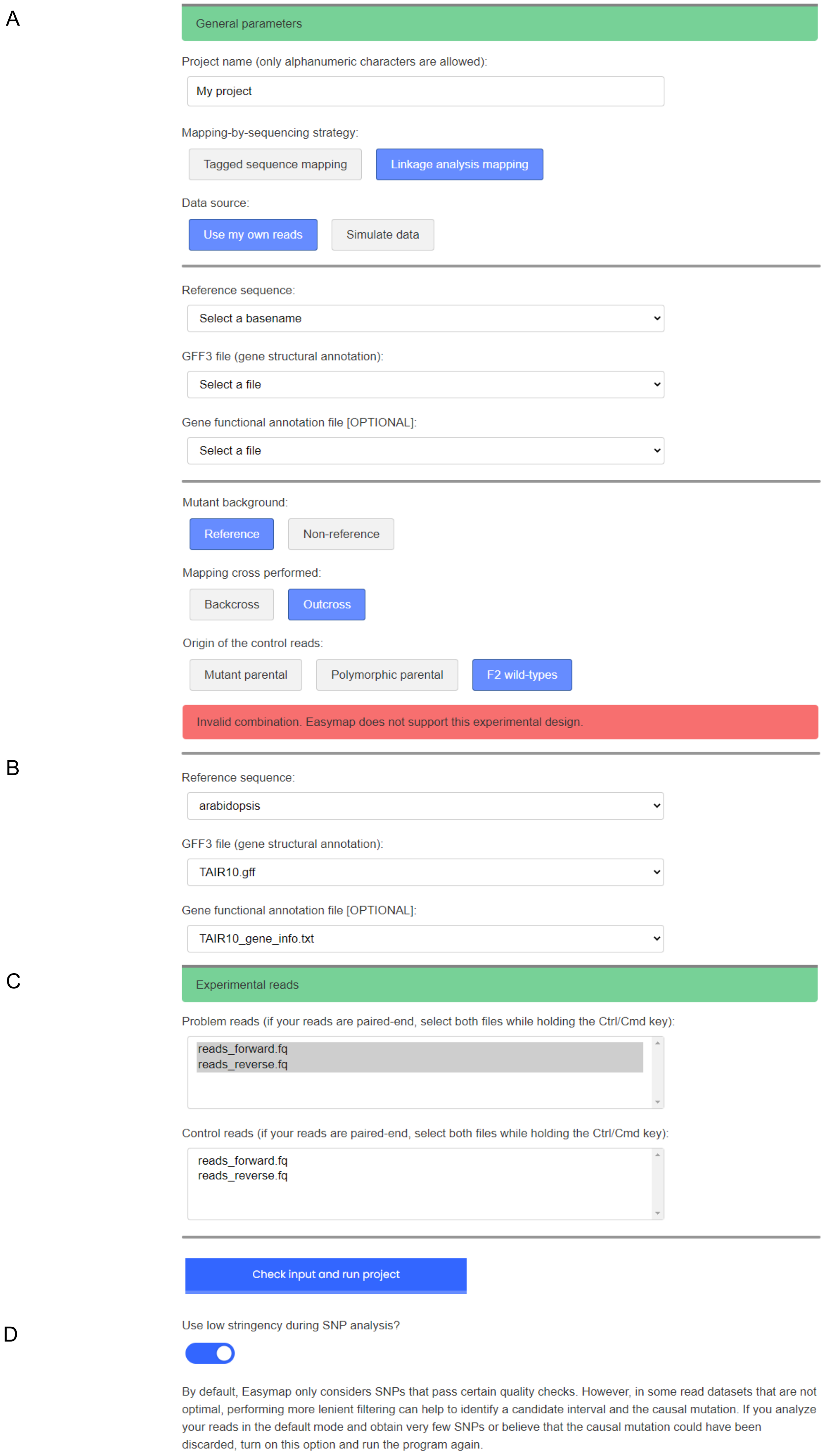
A
General parameters
Project name (only alphanumeric characters are allowed):
My project
Mapping-by-sequencing strategy:
Tagged sequence mapping
Linkage analysis mapping
Data source:
Use my own reads
Simulate data
Reference sequence:
Select a basename
GFF3 file (gene structural annotation):
Select a file
Gene functional annotation file [OPTIONAL]:
Select a file
Mutant background:
Reference
Non-reference
Mapping cross performed:
Backcross
Outcross
Origin of the control reads:
Mutant parental
Polymorphic parental
F2 wild-types
Invalid combination. Easymap does not support this experimental design.
B
Reference sequence:
arabidopsis
GFF3 file (gene structural annotation):
TAIR10.gff
Gene functional annotation file [OPTIONAL]:
TAIR10_gene_info.txt
C
Experimental reads
Problem reads (if your reads are paired-end, select both files while holding the Ctrl/Cmd key):
reads_forward.fq
reads_reverse.fq
Control reads (if your reads are paired-end, select both files while holding the Ctrl/Cmd key):
reads_forward.fq
reads_reverse.fq
Check input and run project
D
Use low stringency during SNP analysis?
By default, Easymap only considers SNPs that pass certain quality checks. However, in some read datasets that are not optimal, performing more lenient filtering can help to identify a candidate interval and the causal mutation. If you analyze your reads in the default mode and obtain very few SNPs or believe that the causal mutation could have been discarded, turn on this option and run the program again.

Figure S2. Easymap graphic interface to design and run a new project.
Example showing the design of a linkage analysis mapping experiment.
(A) Buttons to define the main experimental settings. The selected combination of parameters modifies the downstream sections dynamically. Red messages, as show in the panel, guide the user to avoid mistakes.
(B) Selection of the files contaning information about the reference sequence.
(C) Selection of problem and control reads, previously uploaded.
(D) Option to modify the default behaviour of the program to adapt to non-optimal datasets.
