# Supplementary material for: Easymap: A User-Friendly Software Package for Rapid Mapping-by-Sequencing of Point Mutations and Large Insertions
Source: Front Plant Sci. 2021 May 7;12:655286. doi: 10.3389/fpls.2021.655286 (PMC8143052; doi:10.3389/fpls.2021.655286)
Supplement: Supplementary Data Sheet 2 — Easymap documentation. [file Data_Sheet_2.pdf]

Easymap documentation

I. General description and scope

Easymap is an all-in-one software package that facilitates mutation mapping through the use of next generation sequencing (NGS) reads in model organisms for which a reference genomic sequence is available (e.g., *Arabidopsis thaliana*, *Caenorhabditis elegans*…). It can be used from the command line or through a web graphical interface, in both cases either locally or remotely. Easymap is designed for use with EMS-induced mutants carrying GC→AT transitions and with mutants harboring large insertions such as transposable elements or T-DNAs. To map EMS-induced mutations, Easymap uses DNA-seq or RNA-seq reads from a phenotyped M_2/F_2 mapping population and from a control sample and employs linkage analysis to identify a candidate region. To map large insertions, it requires DNA-seq reads from one or several pooled insertional lines; mapping is performed by capturing genomic sequences flanking the insertions.

II. Availability and installation

Easymap is available under GNUv3 license for UNIX-based operating systems and has been tested in different Linux distributions. For simple installation and environment configuration guidelines you may want to follow the Easymap Quickstart Installation Guide at http://genetics.umh.es/other_files/genetica%20umh%20es/Easymap/Easymap_Quickstart_Guide.pdf. For more detailed information, continue reading this documentation. For Windows users, Easymap can run in the Ubuntu app for Windows 10 available in the Microsoft Store. For Mac OS X users, Easymap can run in a virtual machine running Ubuntu. See section XIII (page 13) for further details on different installation setups. Before installing Easymap, check section X (page 13) to determine whether you need to install any dependencies beforehand. If you are installing Easymap in a shared environment, see section XI (page 13). For the following steps you will need administrator privileges.

Open a Linux terminal, go to the location where you want to install Easymap (the `/home` directory in the example), download the source code, unzip it, and change permissions to allow installation (omit $ sign):

```
$ cd ~
$ wget
http://genetics.umh.es/other_files/genetica%20umh%20es/Easymap/easymap.zip
$ unzip easymap.zip
$ sudo chmod –R 755 easymap
```

Now, to install the program, enter the `Easymap` directory and run the `install.sh` file.

Option 1 (if you only want to use Easymap through the command line):

```
$ cd easymap
$ sudo ./install.sh cli
```

Option 2 (if you want to use Easymap through the command line or its web interface):

```
$ cd easymap
$ sudo ./install.sh server 8100
```

The second argument (8100) corresponds to the port Easymap will be available at to access the graphic interface. Use a port number between 8100 and 8200 that is not already in use; if no port number is specified, Easymap will by default use port 8100, which is typically available on most machines. Installation can take up to 30 minutes on a personal computer; on completion, you will see a message indicating whether the installation was successful or not. If the installation was not successful, please review section X (page 13) to make sure you have all the required dependencies.

To uninstall the program, go to the `easymap` directory and run the `uninstall.sh` script. After that, you can manually remove the whole `easymap` directory.

```
$ cd /home/easymap
$ sudo ./uninstall.sh
```

III. Input files required by `Easymap`

All text files provided to Easymap should be formatted with UNIX line separators (`\n`); this is the case with FASTQ reads obtained directly from high-throughput sequencers or with FASTA and GFF3 files downloaded from biological databases (e.g., http://www.ensembl.org/info/data/ftp/index.html). However, if you edit a file in a Windows OS, it is sensible to check that your file is still readable by Easymap by opening it and inspecting it. If you are using the web interface, click on the “Preview” button that appears to the right of the file name (see section IV, in page 2). File names must contain only alphanumeric characters and no blank spaces.

Mandatory input files

NGS reads of your test sample (and control sample, when required) in FASTQ format (https://en.wikipedia.org/wiki/FASTQ_format). If your reads are single end, you must provide one file (e.g., `sample.fq`), whereas if they are paired end, you must provide two (e.g., `sample_f.fq`, `sample_r.fq`). FASTQ files have quality information associated with each nucleotide call; there are different encodings for this information, and Easymap needs the quality encoding to be “Sanger”. To check whether your reads meet this requirement, and to convert them if necessary, the easiest method is to analyze them with FastQC and convert them with FastQ groomer. Both tools can be found on the public Galaxy server (https://usegalaxy.org/). Most NGS data produced in the past years have Sanger encoding characteristics. Nonetheless, Easymap inspects the input reads at the beginning of each execution and warns the user if their encoding is not Sanger.

To map an EMS-induced mutant, Easymap needs two DNA-seq or RNA-seq read sets (mapping population and control). It is possible to use different types of reads for the different samples (e.g., single-end reads for the mapping population and paired-end reads from the control population). Regarding read depth, Easymap will analyze datasets of any read depth, but values lower than 10× for insertional mutants and 25× for EMS mutants will compromise the accuracy of the results. Above these minimum values, the higher the read depth is, the more accurate and easier the interpretation of the results will be. The program reports the read depth distribution for each sample analyzed. Easymap also checks and reports the quality of the nucleotide calls in the provided reads. Be aware that low-quality calls can also compromise the results. If low-quality calls are at the 5′ or 3′ ends of your reads, consider trimming them with the appropriate software before performing an analysis with Easymap.

Reference genome in FASTA format (https://en.wikipedia.org/wiki/FASTA_format). If your genome has multiple contigs, its sequence can be provided as a single (e.g., `genome.fa`) or multiple FASTA files. In the second case, the file names must have the structure `{basename}.{contig_number}.fa` and share the same `basename` (e.g., `genome.1.fa`, `genome.2.fa`, etc). All the FASTA headers of the contigs must be present in the GFF3 file you provide so that Easymap can link the information in the two files. FASTA and GFF files downloaded from the same databases are normally associated so that the names of the contigs coincide and no manipulation of the files is required. However, if the contig names in the FASTA and GFF files do not match, you must manually reheader the FASTA files. Easymap compares the input FASTA and GFF3 files at the beginning of each execution and warns the user if the headers in the FASTA file are not in the GFF3 file. Use all contigs of the reference genome even if you know the one that contains the mutation of interest; not doing so will increase alignment artefacts. For the same reason, include the reference sequences of organelles such as the mitochondrion (and chloroplast if applicable).

Gene structural annotation of your reference genome in GFF or GFF3 format. These files are available in the main databases of biological sequences and are normally associated with reference FASTA files (http://www.ensembl.org/info/website/upload/gff3.html). If you manipulate GFF/GFF3 files, be sure not to not introduce additional characters such as “ when saving the file, and be sure that the line separators are UNIX-like.

Input files required for specific analyses

FASTA sequence of the insertion. Only required for large-insertion mapping. The file must contain the full sequence of the insertion, but it can also contain additional sequences (e.g., the whole sequence of the vector used to engineer a transgene). If the file contains multiple FASTA headers, Easymap will use the first sequence.

Optional input files

Gene functional annotation of your reference genome. There is no standard format for this information, so Easymap asks for the simplest possible file: A tab-delimited text file with at least two columns, the first being the gene identifiers as found in the gene structural annotation file (e.g., At1g01010 for *Arabidopsis thaliana*), and the remainder containing their description (e.g., gene symbols, functional information, etc.). Except for the first one, columns can have blank records. You can create tab-delimited text files with a spreadsheet editor by saving as “text (tab-delimited) (*.txt)”. Easymap can run without an annotation file, but it then will not include gene functional information in

the mapping report. This file, or a very similar one that can be modified to fit the format specified above, is typically available from model organism databases.

IV. Running Easymap through the web interface

Accessing Easymap

If your machine has Easymap installed (local installation), point your web browser to http://localhost:<port-number> (e.g.: http://localhost:8100). If you are using Easymap remotely (hosted by your institutional servers, in the cloud, or on a virtual machine), you need to use the network IP where Easymap is installed: http://<ip-address>:<port-number> (e.g., http://11.22.33.44:8100). This address will be made available by the machine administrator. In case you forget the selected port number, it is always stored in the text file easymap/config/port.
Manual launch of the server might be required in exceptional cases. If at any time the interface cannot be accessed, use the following command to start up the server manually:

```
$ cd /path/to/easymap
$ bash launch-server.sh <port-number>
```

Uploading files to Easymap

On the main menu, click on **Manage input files**, and then on **Select files**. This will open a browsing window of your operating system that lets you select multiple files at once. Easymap accepts files with the following extensions: .fa, .fq, .fastq, .gff, .gff3, and .txt. If any of your files has a different extension, simply change it to one of the accepted ones (e.g., genome.fasta to genome.fa, sample.fastq to sample.fq). This does not change the file content. The program also takes gzip-compressed files with the extension .gz.

Once you click **Accept**, you will see the files listed ready for upload. Click **Upload files**. Easymap accepts files of up to 200 Gb. When you click on **Upload files** you will see that the upload progress percentage of a file increases and stops at 100%. After that, it may take a few minutes before the file appears listed ready for use under the header "Current files in disk" on the **Manage input files** page. If you upload gzip-compressed files, it will take longer for the files to be ready because they have to be unzipped.

Please do not close or refresh the **Manage input files** page during the file upload, since this may interrupt the upload process. Upload speed to remote installations of Easymap depends on the network, and in some cases, it might not be possible to upload files of several gigabases, even though Easymap features chunked file transfers for that purpose. In those cases, two alternatives to consider are using a local installation and transferring the files to the remote installation through the command line. For manual transfer of input files, they should be placed in the directory "/easymap/user_data".

Managing input files

On the main menu, click on **Manage input files** to see a list of the files currently uploaded to Easymap. To preview a file, click on the **Preview** button next to its name. This will open a new browser tab with the first 1000 lines of the file. This is useful when you want to check the content of very large files (e.g., FASTQ read files) that most machines are unable to open. You can also delete a file from the easymap directory by clicking on the button **Remove from disk** next to its name. This will display a warning message requesting confirmation. Click on **Confirm** to delete the file permanently.

Running a project

A project is an Easymap execution using a given set of files and performing particular tasks based on the user's preferences. On the main menu, click on **Run new project** and choose your preferred files and mapping options. Then, click on **Check input and run project**. This will check the user input and, if valid, will unlock the button **Start project**. Review all your options and click on that button when ready. This will launch the project and redirect you to **Manage projects**, where you will see the new project running. All fields on screen are mandatory except **Gene functional annotation file**. The options are largely self-explanatory, assistance is provided on-screen, and Easymap will check all the choices made by the user. However, for reference, here is a description of each field available:

1. **Project name**: Provide a meaningful name to identify the project. Blank spaces and other non-alphanumeric characters are not allowed as this name will be used to create a directory to store the files related to this project. Easymap will automatically append a timestamp to the project name to make it unique. Use this name to monitor and review the project in the **Manage projects** menu.

2. **Mapping-by-sequencing strategy**: Choose between **Linkage-analysis mapping** for EMS-induced mutation mapping or **Tagged-sequence mapping** for large insertion mapping.
3. **Data source**: Choose between **Use my own reads**, if you want to analyze experimental reads obtained in a high-throughput sequencer, or **Simulate data**, if you want Easymap to simulate reads for you (see section XII).
4. **Reference sequence**: Choose the basename of the genome you want to use as template. Easymap will then automatically select all the files that have the same basename (see section III). If your reference files are not listed in the dropdown menu, check that their names have a "`.fa`" extension.
5. **Insertion sequence file**: Only available for **Tagged-sequence mapping**. Choose the FASTA file that contains the sequence of the insertion that you want to locate in the genome. If your file is not listed in the dropdown menu, check that its name has a "`.fa`" extension.
6. **GFF3 file**: Choose the GFF3 file that contains the structural annotation of your reference genome. If your file is not listed in the dropdown menu, check that its name has a "`.gff`" or "`.gff3`" extension.
7. **Gene functional annotation file**: This field is optional. Choose a file that contains functional information about the genes in the GFF3 file. If your file is not listed in the dropdown menu, check that its name has "`.txt`" extension.
8. **Mutant background**: Only available for **Linkage-analysis mapping**. Choose between **Reference** and **Non-reference**. Pick the former when the genetic background of your mutant is the same as the sequence provided in **Reference sequence**, and otherwise pick the latter. Only certain combinations of **Mutant background**, **Mapping cross performed**, and **Origin of the control reads** are allowed (see Table 1).
9. **Mapping cross performed**: Only available for **Linkage-analysis mapping**. Select between **Backcross** and **Outcross**. Choose the former if you obtained the mapping population by crossing your mutant with its premutagenesis parental (or equivalent), and the latter if you crossed it with a strain polymorphic with its premutagenesis parental.
10. **Origin of the control reads**: Only available for **Linkage-analysis mapping**. Select between **Mutant parental**, **Polymorphic strain**, and **F_2 wild types**. "Mutant parental" is the strain mutagenized with EMS, "Polymorphic strain" is the strain crossed with the mutant in an outcross, and "F_2 wild types" is a bulked group of M_2 or F_2 plants that do not show the recessive phenotype. Select the sample you sequenced to be used as a control during analyses. See Table 1 to know what samples Easymap accepts as control for a particular mapping experimental design.
11. **Use low stringency during SNP analysis?** Only available for **Linkage-analysis mapping**. By default, Easymap considers only SNPs that pass certain quality checks. However, with some non-optimal datasets, more lenient filtering can help identify a candidate interval and the causal mutation. If you analyze your reads in the default mode and obtain very few SNPs or believe that the causal mutation could have been discarded by the program, turn on this option and run the program again.
12. **Test reads**: Only available if you selected **Use my own reads**. Choose the file(s) that contain your reads. If your reads are single end, select only one file; if they are paired end, select two files while holding down the Ctrl/Cmd key. For **Linkage-analysis mapping** mode, these reads must correspond to the M_2 or F_2 recessive mapping population.
13. **Control reads**: Only available if you selected **Use my own reads** and **Linkage-analysis mapping**. Choose the file(s) that contain your reads. If your reads are single end, select only one file; if they are paired end, select two files while holding down the Ctrl/Cmd key. These reads must correspond to the control sample specified in **Origin of the control reads**.
14. **Mutagenesis simulation parameters**: Only available if you selected **Simulate reads**. This field consists of a preformatted JSON string (`{"numberMutations":"0"}`). Replace the values with the desired ones. See section XII for more information.
15. **Recombination and selection simulation parameters**: Only available if you selected **Simulate reads** and **Linkage-analysis mapping**. This field consists of a preformatted JSON string. Replace the values with the desired ones. See section XII for more information.
16. **Contig recombination frequency distributions**: Only available if you selected **Simulate reads** and **Linkage-analysis mapping**. This field consists of a preformatted JSON string. Replace the values with the desired ones. See section XII for more information.
17. **Sequencing simulation parameters**: Only available if you selected **Simulate reads**. This field consists of a preformatted JSON string. Replace the values with the desired ones. See section XII for more information.

If you are over the maximum space allowed for Easymap or the maximum number of simultaneous jobs running, a red warning banner will appear at the top of the page, and the ability to run new projects will be temporarily disabled. This is a safety limit imposed by the administrator of the machine, and can be configured (see section XI).

Manage your projects

On the main menu, click on **Manage projects** to view all the projects stored in the disk from most recent to oldest. For every project, you can see the amount of memory it uses and its status. Project statuses can be “running”, “finished” (all tasks finished without errors), “killed” (the user stopped the project before completion), or “error” (one or more tasks returned an error). The options available for a project depend on its status and are displayed as buttons. Here is a description of these options:

View log file: Points the browser to a live log file. If a project is running, it can be useful to know what tasks are already completed. If a project has finished, this can be used to review what tasks were performed and how long each one took. If a project returned an error, the log file will show what task was responsible and an explanation of the error. An execution can require tens or hundreds of gigabases during certain steps. If the log of an uncompleted project suggests that it is halted, check that you have free space on your disk.
Stop project: Prematurely aborts a running project. A warning message asking for confirmation will appear. Click on **Confirm** to stop a project irreversibly.
Remove from disk: Deletes all files generated by a project. This button is only available if the project is not running. If you want to delete a running project, stop it first. A warning message asking for confirmation will appear. Click on **Confirm** to delete a project irreversibly.
View report: Points the browser to the project report. This button is only available after all the project tasks have been completed. See section VII.1 for a detailed explanation of the mapping reports.

V. Quick start reference to run Easymap through the command line

Place all of your input files in `easymap/user_data`. All files must be unzipped. When you specify your input files in the Easymap command, simply type the name of the file, not the path to it. For more information about the required input files, see section III. Easymap can map both insertions and EMS-derived mutations. Section VI contains a table that describes all the arguments for the Easymap command, but for a quick start, continue reading here.

To map insertions with single-end reads, use this command:

```
./easymap -w ins -n <string> -r <string> -i <file> -p <file> -g <file> -a
<file>
```

where `-w ins` specifies that we want to use a workflow to map insertions, `-n` the project name, `-i` the insertion file, `-r` the reference genome basename, `-p` the test reads, `-g` the GFF3 file, and `-a` the annotation file. The `-a` argument is optional, so if you do not have a functional annotation file, simply omit it. Example:

```
./easymap -w ins -n myProject -r celegans -i Mos1 -p mutReads.fq -g
ceGenes.gff -a ceAnnotation.txt
```

If you have paired-end reads, specify the names of both files separated by a comma:

```
./easymap -w ins -n myProject -r celegans -i Mos1 -p
mutReadsF.fq,mutReadsR.fq -g ceGenes.gff -a ceAnnotation.txt
```

To map EMS-induced mutations with Easymap, let's suppose or this example that (a) we have an EMS-induced mutant that is in the reference background, (b) it was backcrossed to its wild-type parental to create a mapping population, and (c) we have reads from a pool of phenotypically mutant F_2 plants and from the mutant parental. To analyze these reads, use the following command:

```
./easymap -w snp -n myProject -r athaliana -g atGenes.gff -a
atAnnotation.txt -p F2mutF.fq,F2mutR.fq -c wtRefF.fq,wtRefR.fq -ed
ref_bc_parmut
```

where `-c` specifies the control reads and `-ed` the experimental design that was used to obtain the reads. For the list of experimental designs to map EMS-induced mutants supported by Easymap, see section VII.

Easymap comes with a simulator module that can simulate many different experimental scenarios. As an example, if you want to simulate and analyze reads for a virtual mutant with one randomly positioned insertion, first edit the file `/easymap/simulator/sim_parameters.json` to specify the simulation parameters:

```
{
    "SimMut": {
        "NumberMutations":"1"
    },
    "SimRecsel": {
        "ContigCausalMutation":"1",
        "PositionCausalMutation":"30000",
        "NumberRecombinantChromosomes":"100",
        "RecombinationFrequencies": {
            "1":"0,25-1,32-2,34-3,15-4,5-5,2",
            "2":"0,25-1,40-2,23-3,5-4,1-5,1",
            "3":"0,21-1,40-2,29-3,10-4,3-5,1"
        }
    },
    "SimSeq": {
        "Library":"pe",
        "FragmentSize":"500",
        "FragmentSd":"100",
        "ReadDepth":"20",
        "ReadSize":"100",
        "ReadSd":"0",
        "ErrorRate":"1",
        "GCbias":"50"
    }
}
```

`"NumberMutations":"1"` specifies that you want to create one insertion in your reference genome (see section XII for an explanation of each JSON `“name”:“value”` pair). Save the file and run the following command:

```
./easymap -w ins -n myProject -r celegans -i Mos1.fa -g ceGenes.gff -a
ceAnnotation.txt –sim
```

The `-sim` flag turns on the Easymap built-in simulator. Note that now there is no `-p` (test reads) argument.

During the execution of Easymap, all output is redirected to `easymap/user_projects/{timestamp}_project_name/2_logs/log.log`. You can check this file to monitor the execution. An execution can require tens or hundreds of gigabytes during certain steps. If the log suggests that the project halted, check that you have free space in your disk. If the project finishes returning an error,

besides the log, you can check other files under `easymap/user_projects/{timestamp}_project_name/2_logs/`. Once an Easymap job has finished successfully, you can review the results in `easymap/user_projects/{timestamp}_project_name/3_workflow_output/`. See section VII.1 for a complete description of the Easymap report and all the output datasets it produces.

VI. Full list of Easymap command line arguments

`-w, --workflow`<br><br>Type: string (`snp`, `ins`)<br>Required: always	Analysis workflow you want to use. It depends on the type of mutant. Use `snp` for EMS mutants and `ins` for insertional mutants.
`-n, --project-name`<br><br>Type: string<br>Required: always	String containing the name you want to give to the analysis project. After the program has finished, you can find the results in `Easymap/user_projects/{timestamp}_project_name`.
`-r, --reference-sequence`<br><br>Type: string<br>Required: always	Basename of the FASTA file or files that contain the reference sequence. Regardless of whether there are one (e.g., `danio_rerio.fa`) or multiple files (e.g., `danio_rerio.1.fa`, `danio_rerio.2.fa`, …), type `danio_rerio`.
`-i, --insertion-sequence`<br><br>Type: file<br>File format: FASTA<br>Required: only if `-w ins`	Name of the FASTA file that contains the insertion sequence. Accepts files with numbered lines and/or blank lines.
`-g, --gff3-file`<br><br>Type: file<br>File format: GFF3<br>Required: always	Name of the GFF3 file that contains the genome structural annotation. The contig identifiers in this file must match the contig identifiers in the genome reference file(s).
`-a, --annotation-file`<br><br>Type: file<br>File format: custom<br>Required: optional	Name of the file that contains functional information for the genes in the reference genome. The format is custom (see section III).
`-p, --reads-test`<br><br>Type: file<br>File format: FASTQ<br>Required: when `-sim` is off	Name of the FASTQ file(s) containing the reads of your test sample (DNA from bulked F_2 phenotypically mutant individuals). If your reads are paired end, supply both file names separated by a comma without spaces (`readsF.fq,readsR.fq`).
`-c, --reads-control`<br><br>Type: file<br>File format: FASTQ<br>Required: when `–w snp` and `-sim` is off	Name of the FASTQ file(s) that contains the reads of your control sample. See section VII to know what files you can use as control sample. If your reads are paired end, supply both file names separated by a comma without spaces.
`-ed, --experimental-design`<br><br>Type: string<br>Required: only if `–w snp`	String that summarizes the experimental design followed to obtain the read datasets you provide to Easymap. See Table 1 to know what string matches your experimental design.
`-ls, --low-stringency`<br><br>Type: flag	Only applicable for `–w snp`. By default, Easymap considers only SNPs that pass certain quality checks. However, in some read datasets that are not optimal, performing more lenient filtering can help to identify a candidate interval and the causal mutation. If you analyze your reads in the default mode and obtain very few SNPs or believe that the causal mutation could have been discarded, turn on this option and run the program again.
`-sim, --simulate-reads`<br><br>Type: flag	Turns on Easymap built-in function to simulate the data for a mapping experiment. See section XII for details of how to specify the simulation parameters.
`-u, --usage`<br><br>Type: flag	Shows where to find help to run Easymap through the command line interface.

VII. Experimental designs supported by Easymap to map EMS-induced mutants

Easymap requires two read datasets for mapping of EMS-induced mutations. The first corresponds to a pool of phenotypically mutant individuals from an M_2 generation or from the F_2 generation of a mapping cross. The second corresponds to a control sample, and depends on the background of your mutant and the source of the mapping population. Since Easymap includes a splice-site aware aligner, both datasets can come from a DNA-seq or RNA-seq sample. See Table 1 to know which samples can be used as control in each case.

Table 1.- Experimental designs for EMS-induced mutation mapping supported by `Easymap`

Genetic background of the mutant	Source of the mapping population	Test sample	Control sample	Argument value `-ed` / `--experimental-design`
The same as the reference sequence provided	M_2 or backcross F_2	DNA from a pool of M_2/F_2 phenotypically mutant individuals	Mutant parental	`ref_bc_parmut`
			Pool of M_2/F_2 phenotypically wild-type individuals	`ref_bc_f2wt`
	Outcross F_2		Mutant parental	`ref_oc_parmut`
			Polymorphic strain	`ref_oc_polstr`
Other than the reference sequence provided	M_2 or backcross F_2		Pool of M_2/F_2 phenotypically wild-type individuals	`noref_bc_f2wt`
	Outcross F_2		Mutant parental	`noref_bc_parmut`

VII.1. SNP selection and filtering parameters

Easymap selects high-confidence polymorphisms for mutation mapping according to the following criteria: (1) SNPs with quality values as defined by the variant-calling pipeline over 120 for the high stringency mode and over 30 for the low stringency mode. (2) SNPs with reasonable read depth, avoiding excessively low or high values as compared with the overall average read depth. For datasets with an average read depth (RD) over 25, we filter out SNPs with an RD lower than 15, to ensure the use of high-quality polymorphisms for the mapping steps. For datasets with an average RD below 25, we filter out SNPs with an RD below 10 for the same purpose. For datasets with an average RD over 40, we filter out SNPs with an RD greater than three times the average RD, while for datasets with an RD under 40, we filter out SNPs with an average RD over 100. (3) EMS-type mutations for mutants in a reference genomic background backcrossed with the mutant parental line. These arbitrary thresholds have proven to be effective in the selection of high-confidence polymorphisms for mutation mapping, avoiding conflictive SNP.

For candidate mutation reporting, Easymap applies the following filters: (1) SNP exclusive to the test sample, (2) contained within the candidate region, (3) with an allele frequency higher than 0.8 and (4) EMS-compatible mutations.

VII.2. Mapping algorithms general overview

Easymap identifies candidate regions from different sources of data belonging to different experimental designs. The pipeline first divides the genome into overlapping windows, the size and degree of overlap varies depending on the experimental design. Next, the SNPs allele frequencies (AF) are averaged in each window and optionally transformed in a Boost value if the experimental

design includes an outcross (Sun H., Schneeberger K., 2015). Average AF or Boost values are then revised and corrected by comparing adjacent windows to reduce noise and randomly generated peaks, using a weighted average approach that takes into account up to 6 adjacent windows for correction. Easymap analyzes the revised AF in each window to select the highest value as the window that most likely contains the candidate mutation, which will define the phenotypically-selected genomic position or center of the candidate interval. Finally a candidate interval of 4, 10 or 20 Mb is defined depending on the size of the input genome, and the mutations within the candidate interval are reported as candidates.

VIII. Explanation of the Easymap output report

The Easymap output report consists of an HTML document whether you executed Easymap through the command line or through the web interface. To view the report of a project generated through the command line, go to `easymap/user_projects/{timestamp}_project_name/3_workflow_output/report.html`. To view it in the web interface, go to **Manage projects**, locate the desired project, and click on **View report**. The report file is only available when the project status is "finished". The report contains all relevant information for each project organized in different sections. To create a PDF or paper copy of the report, simply use the printing menu of your browser, which will print an optimized version.

VIII.1.- Linkage analysis mapping report

Section 1: Run summary

This section contains general information about the project, including the name of the input files and the options selected for the mapping analysis.

Section 2: Input data quality assessment

A few checks on the quality of the provided data are performed in order to assess the reliability of the mapping results:

- **Test and control sample read depth distributions**: These graphics plot the distribution of read depth frequencies found during the alignment of the reads to the genome and gives a visual estimation of the average read depths in the test and control samples. Samples of lower average read depth will result in less reliable mapping results.
- **Reads quality assessment**: A box-plot representing the variation of the quality of the reads along the read length. Low-quality reads will hinder the mapping analysis.

Section 3: Mapping analysis overview

In this section, all input contigs are displayed along with the polymorphisms used for mapping the causal mutation and the linear descriptor of the allele frequency. The graphs plot the position of each polymorphism in the contig (*x* axis) and the allele frequency of the non-reference allele (*y* axis). The candidate region determined by the mapping analysis is highlighted in pink, and polymorphisms from the test F_2 sample and the control sample are drawn in blue and orange, respectively.

Section 4: Candidate polymorphisms overview

The contig containing the candidate region is displayed along with all of the polymorphisms in the test sample that pass the quality checks. The window that contains the candidate mutations is highlighted, and the selected chromosomic position is represented as a dashed line. This window only includes polymorphisms with an allele frequency >0.8. An additional image zooms into the candidate window displaying the final candidate polymorphisms, filtered to include only typical EMS mutations.

Section 5: Candidate region analysis

This section contains a table summarizing information about each candidate polymorphism. Each row corresponds to a polymorphism and contains the following information:

- **ID**: Numeric identifier assigned by Easymap to each candidate. It can be used to correlate information within the mapping report.
- **Genomic position**: The contig and the absolute position of the polymorphism.
- **AF**: Frequency of the non-reference allele in the test F_2 sample.
- **Distance to peak (DTP)**: Distance between the polymorphism and the selected genomic position determined by Easymap. Polymorphisms to the left and right sides of the selected position have negative and positive distances, respectively.
- **Nucleotide (Ref/Alt)**: The reference and alternative alleles of the polymorphism.

Gene (gene element): Gene identifier, as given in the input GFF file, and element of the gene that contains the polymorphism (coding sequence [cds], intron, promoter, etc). Easymap's variant analyzer reports putative splicing signal modifications in Linkage analysis mapping mode: 2 nucleotides (nt) in the intron borders and 1 nt in the exon border. Other intronic regions, such as branching sequences, are not analyzed.

Amino acid (Ref/Alt): When a polymorphism results in a change in a protein sequence, the reference and alternative amino acids are given.

Additional information can be seen by clicking on the “extended information” link below the table; this downloads a text file with all the available information regarding the candidate polymorphisms. Furthermore, a file containing the information about all of the polymorphisms in the F_2 test sample can be downloaded by clicking on the “all variants” link. These files contain the following additional fields:

quality: Phred-scaled quality score for the polymorphism as given by the variant calling pipeline.

ref_count: Number of reads containing reference allele of the variant.

alt_count: Number of reads containing alternative allele of the variant.

hit: Indicates if the mutation is located within a transcription unit (tu), in a regulatory region (rr), or in intergenic regions (nh, no hit).

mrna_start, mrna_end: Genomic coordinates of the transcription unit containing the mutation.

strand: Indicates the orientation of the gene within the contig.

gene_funct_annot: Gene functional annotation. If a functional annotation file is provided, this column contains information regarding the gene that contains the variant.

f_primer, r_primer: Forward and reverse primers designed for genotyping purposes. The amplified fragment should have a length of around 800 nt, with the polymorphism at a distance of approximately 300 nt from the forward primer.

tm_f_primer, tm_r_primer: Melting temperatures of the forward and reverse primers. Melting temperatures range between 60 and 64°C.

upstream, downstream: 50 nucleotides of DNA sequence upstream and downstream of the position of the variant.

Easymap may fail when choosing the selected genomic position; in this case the user can review the data to determine the most probable position for the mutation. With this in mind, if no candidate variants are detected during the analysis, this section contains a link to the file with information regarding all variants in the genome.

Section 6: Candidate variants

This section contains a list of the candidate mutations affecting gene open reading frames. An image is generated representing each candidate gene: exons and untranslated regions are shown as boxes (dark and light blue respectively) connected by introns shown as lines. A 250-nt putative promoter region is included as a dashed line. The position of the variant is indicated with a red arrow accompanied by the nucleotide change and the amino acid change (when applicable). Finally, the image contains a scale bar to be used as a visual reference. The image is followed by a table containing relevant information about the variant.

VIII.2.- Tagged sequence mapping report

Section 1: Run summary

This section contains general information about the project, including the names of the input files and the options selected for the mapping analysis.

Section 2: Input data quality assessment

A few checks on the quality of the available data are performed to assess the reliability of the mapping results:

Test and control sample read depth distributions: These graphics plot the distribution of read depth frequencies found during the alignment of the reads to the genome and give a visual estimation of the average read depths in the test and control samples. Samples of lower average read depth will result in less reliable mapping results.

Reads quality assessment: A box-plot representing the variation of the quality of the reads along the read length. Lower-quality reads will hinder the mapping analysis.

Section 3: Mapping analysis overview

3.1 Genomic overview: A representation of the input contigs with the positions of all detected insertions marked with red triangles.

3.2 Insertions summary: This section contains a table with the summarized information about all insertions found in the genome. Each row corresponds to an insertion and contains the following information:

Ins: Numeric identifier given to each insertion.

Contig: Input contig that contains the given insertion.

Position: Location of the insertion within the contig.

Gene (gene element): Gene name as given in the input GFF file and element of the gene that is interrupted by the insertion (coding sequence [cds], intron, promoter, etc).

Wt amino acids: Number of wild-type amino acids conserved in the mutant protein.

Additional information can be seen by clicking on the "extended information" link, which downloads a text file with all the available information regarding the insertions. This file contains the following additional information (omitting fields already explained in the linkage analysis mapping report section):

5_end_ins, 3_end_ins: Reconstructed sequences for the 5′ and 3′ ends of the insertion as found in the mutant genome. The sequences are used to generate primers for genotyping the insertion.

insertion_primer_5, insertion_primer_3: Primers contained within the 5′ and 3′ ends of the insertion designed for genotyping purposes.

tm_insertion_primer_5, tm_insertion_primer_3: Melting temperatures of the described primers. Melting temperatures range between 60 and 64°C.

Section 4: Detected insertions

This section contains detailed information about each insertion, starting with histograms that visually summarize the mapping information. The histograms plot the read depth versus genomic position of the reads that support the detection of each insertion. When paired-end reads are available, two histograms are shown: (1) Flanking unpaired alignments: Reads that cannot be aligned to the insertion sequence, but whose mates can; they surround the position of the insertion site in the genome; (2) Flanking local alignments: Reads that are aligned locally to the insertion sequence are then realigned to the genome sequence, marking the position at which the insertion event has occurred. When single-end reads are used, only the Flanking local alignments histogram will be available.

If a given insertion interrupts one or more genes, the information about each one is listed below the histograms, starting with a graphical representation of the gene in which the location of the insertion is marked with a red triangle. The image is followed by relevant information about the insertion, such as its position, the functional annotation (when available), the genotyping primers, and the genome sequences flanking the insertion site.

IX. Content of the projects directory

A report file is automatically created whether the program is run through the web interface or the command line. When a project is run through the command line, the report file must be accessed and opened from `easymap/user_projects/{timestamp}_project_name/3_workflow_output/report.html`. The report contains all relevant information for each project; however, additional files such as individual image files and tab-delimited text files can be accessed in the projects directory through the web browser, the command line, or the operating system file manager. The projects directory contains the following three folders:

1_intermediate_files: Contains intermediate files generated during the execution of a mapping analysis. Raw data and large intermediate files, such as SAM, BAM, or VCF files, are automatically deleted during the execution of the program to save storage space.

2_logs: This folder stores log files automatically produced by third-party software such as bowtie2 and SAMtools, as well as the Easymap log file for the given project (log.log).

3_workflow_output: Contains the report file (report.html), the images shown in the report, and additional images that might be of interest. This folder also contains the tab-delimited text files referenced in the report file (`candidate_variants.txt`, `candidate_variants_total.txt`). The names of all files in this folder are self-explanatory.

X. Appendix A: Dependencies needed to run Easymap

All third-party software needed for the analyses is included in Easymap; however, a few basic Linux packages are required for the installation of Easymap. To prepare the system for the installation, please follow the next steps.

In Ubuntu 20, run the following commands:

```
$ sudo apt-get update
$ sudo apt-get install build-essential zlib1g-dev libbz2-dev git wget tar zip liblzma-dev libncurses5-dev libncursesw5-dev libssl-dev make -y
```

In Ubuntu 18, run the following commands:

```
$ sudo apt-get update
$ sudo apt-get install build-essential zlib1g-dev libbz2-dev liblzma-dev libncurses5-dev libncursesw5-dev libssl1.0-dev wget tar zip git
```

In older versions of Ubuntu (tested in 16.04 and 14.04), run the following commands:

```
$ sudo apt-get update
$ sudo apt-get install build-essential zlib1g-dev libbz2-dev liblzma-dev libncurses5-dev libncursesw5-dev libssl-dev wget tar zip git
```

In RPM-based Linux distributions (Tested in Red Hat) run the following commands:

```
$ sudo yum groupinstall "Development Tools"
$ sudo yum groupinstall "Development Libraries"
$ sudo yum install wget zlib-devel bzip2-devel ncurses-devel ncurses openssl-devel
$ sudo yum install -y xz-devel
$ sudo yum install curl
$ sudo yum install curl-devel
```

XI. Appendix B: Installing Easymap in a shared environment

The installation script sets unrestricted permissions recursively in the `easymap` directory. Change them manually after the installation if you need more restrictive permissions. If you are installing Easymap for other users and want to limit the resources available to them, edit the file `easymap/config/config`. Further instructions can be found inside the file.

The installation script installs `python2.7` locally under the `easymap` root directory to make sure it is available for Easymap without interfering with other installations that might already be on the machine. It also installs Virtualenv locally for the same reason. After that, it creates a virtual environment named `easymap-env` to isolate any other python libraries such as Pillow.

To make the Easymap web interface easily available for most users, the installation command `./install.sh server <port-number>` starts the python CGIHTTPServer in the background and modifies `/etc/crontab` so that the server is also started after each reboot. If you want to avoid that, use `./install.sh cli`. Easymap includes an additional web interface in `easymap/web_interface_PHP` based on a HTML-Javascript-PHP stack easily deployable in servers such as Apache2 that have PHP enabled.

XII. Appendix C: How to simulate data with Easymap

To simulate data through the command line, include `--simulate-data/-sim` in your command and specify the simulation parameters by editing the file `easymap/simulator/sim_parameters.json`. The fields included in the file can also be edited through the graphical interface. Do not change the name of the file. A simulation of an insertional mutant consists in the creation of a mutant FASTA and high-throughput FASTQ reads. A simulation of an EMS mapping population and a control sample also involves the creation of recombinant FASTA files.

`"NumberMutations"`	Specifies the number of mutations in the mutant. The reference sequence provided with `-r` is used as template to create a mutant version. If `-w ins`, the insertion sequence provided in `-i` is used as insert, and the number of insertions is limited to 1 per megabase of reference genome. If `-w snp`, the number of point mutations is limited to 100 per megabase of reference genome. Both insertions and point mutations are generated in random positions.

"ContigCausalMutation"<br><br>Is ignored if `-w ins`	Integer that specifies the contig number that will have the causal mutation.
"PositionCausalMutation"<br><br>Is ignored if `-w ins`	Integer with the position, in the contig specified with "ContigCausalMutation", of the causal mutation defined in base pairs. Must be between 1 and the length of the contig. If `-w snp` and the position does not correspond to a G or C, Easymap will use the closest G or C instead to generate an EMS-like mutation.
"NumberRecombinantChromosomes"<br><br>Is ignored if `-w ins`	Integer that represents the number of haploid F_2/M_2 recombinant genomes to create.
"RecombinationFrequencies"<br><br>Is ignored if `-w ins`	Recombination frequency distribution of each contig provided in `-r`. It must contain a "name":"value" pair for each contig in the reference sequence. "value" is a list of `event,frequency` pairs, where event is the number of crossover points per contig and frequency the percentage of contigs in a recombinant contig population. `event,frequency` pairs are separated by a dash. "name":"value" pairs are separated by commas.
"Library"	Type of fragment library for sequencing. Choose between `se` (single-end library) and `pe` (paired-end library).
"FragmentSize"	Average size of the library fragments. Must be an integer equal or greater than the read length specified in "ReadSize".
"FragmentSd"	Standard deviation of the size of the library fragments. Must be a positive integer.
"ReadSize"	Average size of the reads. Must be a positive integer.
"ReadSd"	Standard deviation of the size of the reads. Must be a positive integer.
"ReadDepth"	Number of times in average that each nucleotide in the reference sequence is read. Must be a positive integer.
"ErrorRate"	Integer in the interval [0–5] that represents the base-calling error rate in percent.
"GCbias"	Integer in the interval [0–100] that represents the GC content bias strength of the library. Setting this to >0 penalizes the creation of fragments with non-neutral GC content. The less neutral the GC content of a genomic sequence, and the bigger the strength value set by the user, the less probable it is that the sequence is present in the set of reads created.

XIII. Appendix D: Other installation setups

This section includes some helpful notes for the installation of Easymap in different setups. Please note that the performance of virtual machines and apps is reduced compared to that of a direct installation of an OS, so for low-performance machines these options are unadvisable.

Installation in Windows 10 Ubuntu app. After installing Ubuntu from the Microsoft Store (search for "Ubuntu 18.04 LTS"), you may be prompted to enable the "Windows Subsystem for Linux" feature of the Windows 10 OS. To do so, run the Windows 10 PowerShell as administrator (you can find it at the start menu), type the following command on the PowerShell, hit enter and then restart your system:

```
Enable-WindowsOptionalFeature -Online -FeatureName Microsoft-Windows-
Subsystem-Linux
```

Once the Ubuntu app is working, Easymap can be installed by running the installation script as explained in section II or in the Quickstart Installation Guide. Make sure the dependencies specified in Appendix A are installed by running the commands provided. Automated startup of the Easymap dedicated server does not work within this environment; in order to access the web interface, the server needs to be started up manually after each reboot with the following commands:

```
$ cd /path/to/easymap
$ bash launch-server.sh <port-number>
```

The interface is accessed at http://localhost:<port-number> and can only be used locally; remote access to the server is restricted by the system.

Installation in Amazon EC2. For remote installation in a cloud computing service, the remote instance should be setup to allow HTTP access. In Amazon EC2, the recommended Amazon Machine Image (AMI) is Ubuntu Server 18.04. During setup, in the "Configure Security Group" tab, click on "Add Rule", choose the "All traffic" option, and change the "Source" option to "Anywhere". Once the instance is set up, Easymap is installed as explained in section II. To access the web interface, point your browser to the Public DNS address assigned to the instance, adding ":<port-number>" at the end.

Installation in Oracle Virtualbox. Oracle Virtualbox is a free software for running virtual machines available for Windows and Mac OS. Ubuntu Server can be downloaded for free from https://releases.ubuntu.com/18.04.4/ubuntu-18.04.4-live-server-amd64.iso and installed within the Oracle Virtualbox environment. This link contains general instructions for the installation of the Ubuntu OS in a Virtualbox virtual machine: https://linuxhint.com/install_ubuntu_virtualbox_2004/ . During the configuration of the virtual machine, the network should be set as "bridged network" so that the web interface can be accessed later. A minimum of 4 Gb of RAM and a fixed-size virtual hard drive of at least twice the size of the reads to be processed is recommended for the virtual machine. Once the virtual machine is running you will have access to a Ubuntu terminal, from where Easymap can be installed as explained in section II or in the Quickstart Installation Guide. After installation, you can access the Easymap web interface using the virtual machines IP address from your web browser at: http://<ip-address>:<port-number>. To find your IP address, you can run the following command and select the address from the "inet" field:

```
$ ip addr show
```

Output example: inet 11.22.33.44/55 brd 10.1.31.255 scope global dynamic
IP address: 11.22.33.44

XIV. Appendix E: Modifying Easymap workflows

Easymap was designed to be as user-friendly as possible, hardcoding most of the parameters used during the alignment of the reads, the variant-calling or posterior filtering steps, to save the user from having to set any complex parameters. If you are an advanced user and wish to adapt Easymap workflows to any specific need or to add steps to the analyses, you can find the main workflows used by Easymap in the folder /easymap/workflows. In addition, workflow-ins.sh contains the code used for Tagged-sequence-mapping, and workflow-snp.sh contains the instructions for linkage analysis mapping. The code is written in commented and well organized blocks to easily find and isolate any specific part of the analysis. The threads variable in workflow-snp.sh can be modified to allow multithreading during the compatible steps of the analyses. You can also modify the parameters used for the read alignment and variant calling steps in the Samtools and BCFtools command. Furthermore, the window size and distance between windows used by the mutation mapping script map-mutation.py can be modified by changing the arguments -window_size and -window_space in workflow-snp.sh for any specific experimental design.
